# Supplementary material for: Comparison of Robotic and Conventional Unicompartmental Knee Arthroplasty Outcomes in Patients with Osteoarthritis: A Retrospective Cohort Study
Source: J Clin Med. 2021 Dec 31;11(1):220. doi: 10.3390/jcm11010220 (PMC8745819; doi:10.3390/jcm11010220)
Supplement: Supplementary file 1 [file jcm-11-00220-s001.zip › jcm-1499935-SI.pdf]

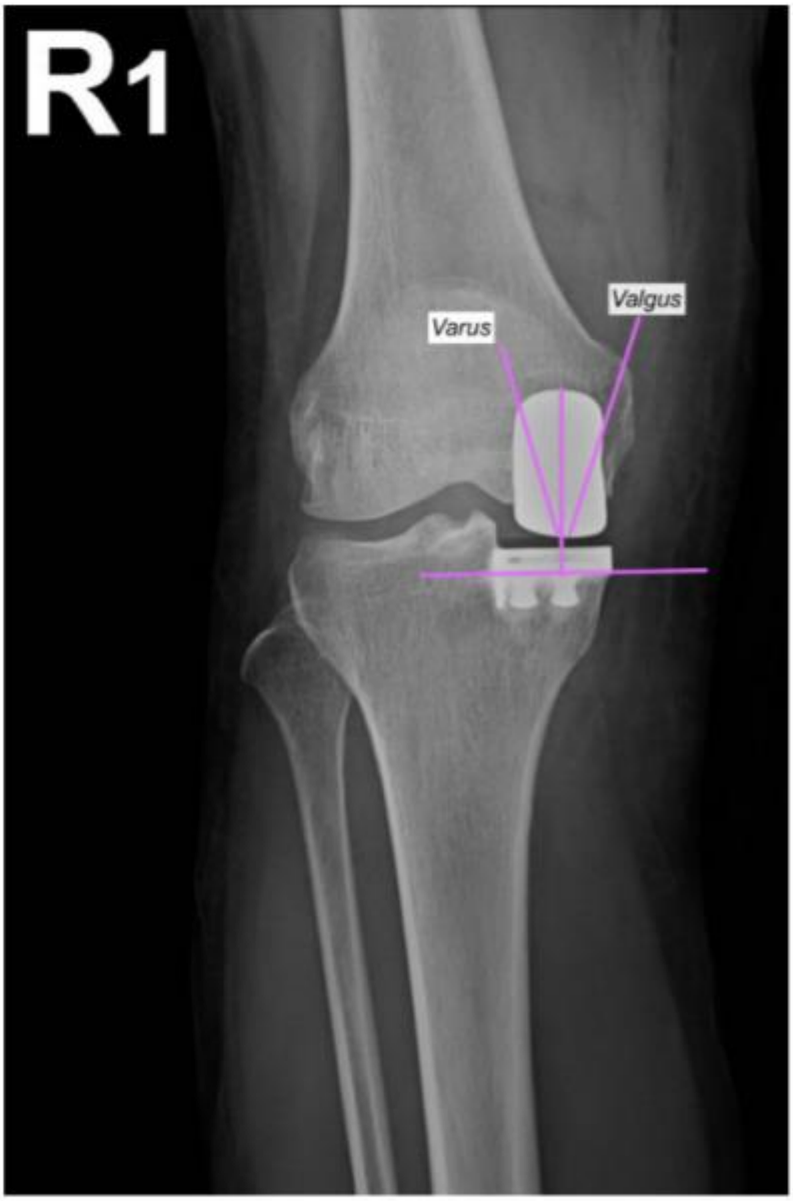

Figure S1. Assessment of the implant alignment grossly based on the radiograph

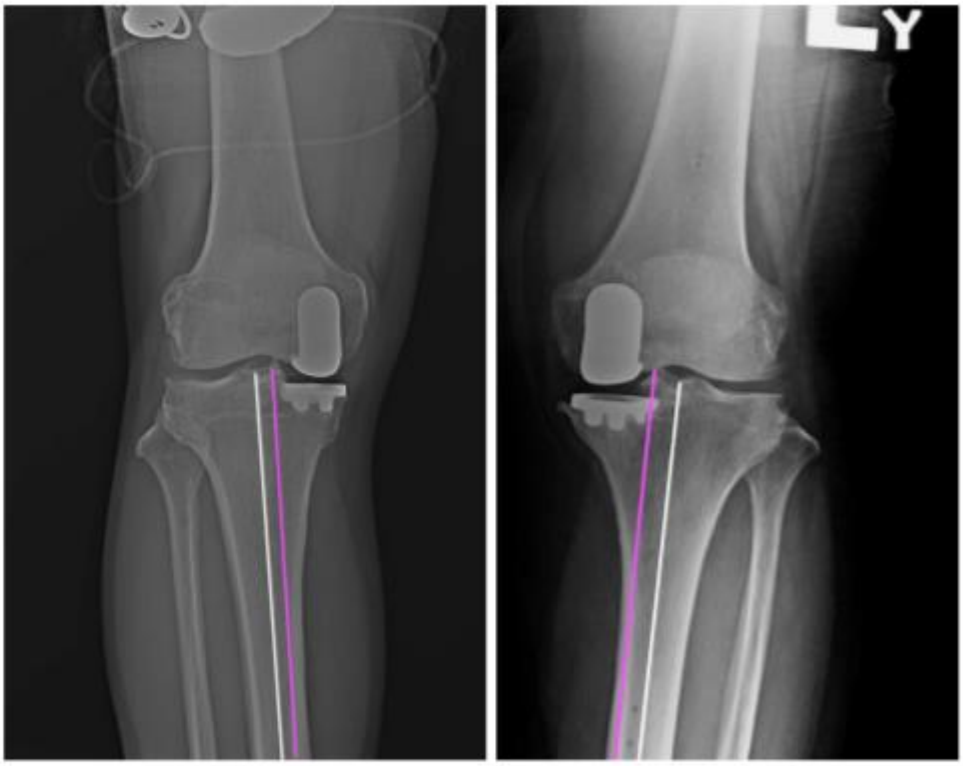

Figure S2. Assessment for the preservation of tibial eminence based on the radiograph

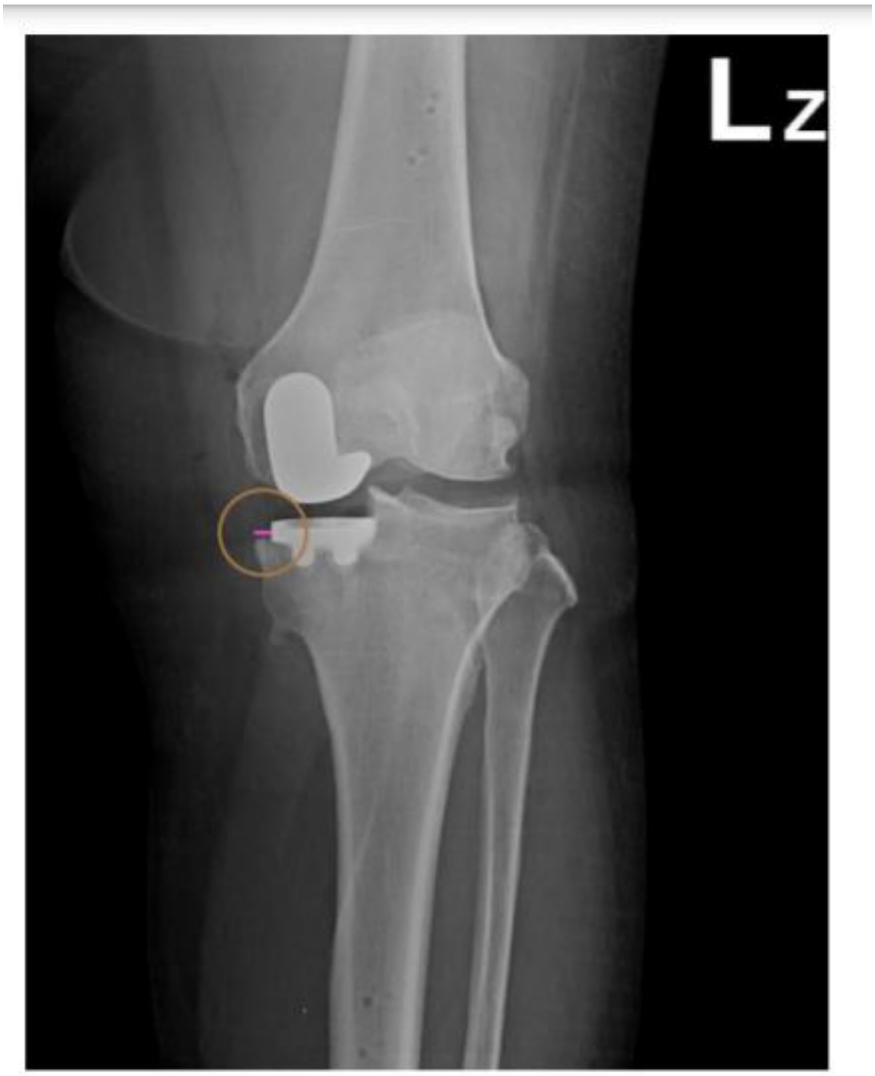

Figure S3. Assessment of the implant positioning as overhang or normal.
